# The Evolution of the Modern Hospital

**Published:** 1915-04-03

**Authors:** B. Burford Rawlings


### THE EVOLUTION OF THE MODERN HOSPITAL.*

### V.—Medical Education, Administration, and Finance *(continued)*.

By B. BURFORD RAWLINGS.

That the relations of lay and medical work in hospitals obtain some, if insufficient, attention, was evidenced when the authorities of the King's Fund appointed a committee to report concerning the claims of the schools upon the hospitals. The committee was not equipped adequately for an investigation of the whole subject, and as a matter of fact the field of its deliberations was strictly limited. The inquiry how far the schools are entitled to look to hospital funds for direct aid towards expenditure was considered and answered, but no mandate was given and no attempt was made to enter upon the much wider question concerning the effect upon hospital finance produced by the association of education with the medical treatment of the poor. Apart from the principle involved, the sum openly diverted from the hospital to the school never has been of first importance. In no hospital has it exceeded a trifling percentage of the total expenditure. It is when we come to consider the influence of the connection between school and hospital, not less upon the receipts than upon the general expenditure, that we begin to realise the true proportions of the questions involved. The fact that a period of aggravated financial difficulty has synchronised with the increasing demands for a tighter professional grip upon hospitals is suggestive.

### The Dues of "Honorary" Service.

Upon all sides it is asserted that appeals have ceased to be effective; the help of the central funds has alone saved many of the institutions from disaster. Moreover, analysis of the success which has attended the King's and the Sunday Funds shows that it is but little due to popularity, and just as each individual hospital has always depended in chief measure upon the good offices of a small band of supporters, so these important and benign organisations owe the greater part of their power of usefulness not to the general support they deserve, but to the munificence of a few enthusiasts. Indigence, whether of men or institutions, is a great evil, because it is fatal to liberty and palliates failure. In the case of the hospitals, it is inimical to capable government. Not only is there an emasculating sense of dependence upon casual gifts, but poverty (it could not be policy) has decreed that the most important workers must go unremunerated. That the services of the medical and surgical staff obtain indirect and unacknowledged return does not affect this view of the matter, unless it be to aggravate the consequent evils. A position upon the staff is termed "honorary," and the dues of honorary service are exacted. "We are busy men, and you must take what we can give" was the frank reply of a physician, eminent both in practice and teaching, when the subject of punctual attendance was under discussion. The case could not be put more clearly or more truthfully. Practically, the staffs of the several hospitals make up the whole body of consultants, and these gentlemen, in addition to the care of many thousands of in-patients and vast bodies of out-patients, are concerned with the education of students and must satisfy the demands of large private practices. It follows that the time devoted to the individual patients of the hospital is often in the inverse ratio of the eminence of the practitioner, and although the rules may be explicit, for a managing body to insist upon their observance is out of the question. An honorary staff's utmost acknowledgment of obligation is the minimum demanded by professional etiquette, and occasions are rare when professional etiquette can be invoked in support of unprofessional authority.

### The Residents' Position.

It would be unfair to require of a physician a performance of gratuitous service at the cost of a fee awaiting him elsewhere. He is debtor to the hospital, but he also owes something to himself. The custom which sanctions the abandonment of the hospital visit at the call of a private patient would be incapable of justification under sounder conditions of service, but it is native of the system, and we may well marvel that under the conditions which obtain occasions are so frequent when the observer is impelled to admiration of the patient and prolonged consideration given to hospital inmates. Doubtless the advantages of the hospital connection would compensate for the surrender of many fees, but the time devoted to the hospital is regarded as a free gift, and the physician measures it not always in accordance with hospital rule or hospital necessity. With the time at his disposal it may be impossible to go deeply into the details of every case, and if he be an active teacher and lecturer, his house physician, whose business it is to see that clinical material is forthcoming, will sort out the patients for his chief like a hand at cards, the trumps being the cases which supply the best texts for comment and demonstration before the crowd of students and medical visitors who follow him on his rounds, or for inclusion in his next book. In these circumstances the treatment of many patients passes into the hands of residents immature both in years and attainment, whose unaided judgment must often decide vital questions. Reference to the authority already quoted corroborates this assertion. "In many hospitals," he says, "there is a great deficiency in not having permanent officers of long experience to discharge some of those duties which might be called for at any hour of the day or night and are at present left too much in the hands of young gentlemen who . . . have not the practical experience of

* Previous articles: February 20, March 6, 13, 20, and 27.

medical men in general practice." These words, although not of recent utterance, need no qualification. They represent the case as it is.

Nobody understands better than the holders of resident offices that their association with the hospital is fleeting and educational. They are instinct with the spirit of the school to which in their view the hospital is, first of all, an illustrative and nutrimental adjunct. The best function of a hospital is to feed its school. The institution as a whole makes no demand upon their attention or their sympathy, and its philanthropic responsibilities are as remote from their purview as the problems of finance or the ministrations of the chaplain. No condemnation is too severe for a system which allots positions of responsibility and almost uncontrolled authority as prizes to students, whose disqualifications of youth and inexperience never come under the mellowing influences of time, but are reproduced by automatic process with the regularity of the calendar.

Not very long ago the newspapers told how a dying woman, taken by her friends to a West End hospital, was seen by a house physician, who failed to appreciate the gravity of her condition, and was sent away again upon the plea that a bed was not available. It so happened that the woman bore the recommendation of somebody who had been concerned only a few weeks before in the allotment of a very large benefaction to the hospital funds. When the facts were published, attention fixed itself upon this circumstance to the exclusion of all else, the real lesson of the accident being overlooked; and so firm is the professional grip upon many of our leading journals that comment was refused, and no letter upon the subject, surely of great public importance, succeeded in obtaining a place.

Yet none would deny that house physicians and house surgeons in their relations with the patients are kindly and attentive; in the discharge of their duty to members of the staff they are scrupulous and painstaking. They entertain no illusions; they do not expect to be reckoned philanthropists. They are aware that their term of residence furnishes the climax of their career as students, and they make use of their opportunities accordingly. When in actual attendance upon a patient whose life perhaps hangs upon their efforts, their best qualities shine out, and no trouble is too great, no exigency too exacting. Only a few of the residents and clerks who pass through the hospitals aspire to become simple scientists, and to take their places among the brilliant but chill company whose minds are directed to illuminate the frozen ways. Mostly they are content with a less exalted but not less useful rôle, and are eventually numbered with the best loved and best trusted of mankind whose claims to gratitude are among the abiding memories of our homes. A suggestion often repeated is that the educational uses to which the hospital ward is put help to ensure accuracy in diagnosis. Doubtless, the voluminous notes taken by the clerks are the results of careful and exhaustive examinations compassing the minutest details, and the consultant who bases his treatment upon them would look severely upon mistake or want of precision, but uniformity of excellence is impossible, and unfortunately ward etiquette is not conducive to rectification of error. So strictly enforced are the restraints of medical discipline that few sisters or nurses would dare to correct a resident when reporting to the physician on a matter of fact, no matter how flagrant his mistake.

The connection of the schools offers opportunities to unfriendly critics, and the knowledge that the sensitive minds of many donors regard it as injurious to the pure benevolence of hospital work is utilised to create prejudice. The Anti-Vivisection Societies are not slow to fasten upon the fact. Unhappily, vivisection and medical education are allied too closely in the public mind, and the notion has been fostered that hospitals are involved in the practice; most unfairly, because hospital authorities have no part in the management of the schools and are never asked whether they approve or disapprove of vivisection. The fact that the medical curriculum of London University requires the presence of students at vivisectional demonstrations became generally known when the reports were published of proceedings in connection with a certain case in the Law Courts. Public feeling was greatly shocked by the revelation, and without doubt hospitals suffered. That hospital managers have no part in the arrangements of the schools is not realised in respect of fact or bearing, and if there be any truth in the assertion made two or three years ago by anti-vivisectionists that acts of vivisectional experimentation upon animals had increased from 481 in 1876 to 88,634 in 1908 it is certain that science must suffer from its excesses; and if the opposition of prejudiced and perhaps ignorant people ultimately prevail and all vivisection is forbidden, the scientists will have done more to bring about that result than the utmost efforts of their opponents.

The bare suggestion of research is repulsive to many of those upon whom the hospitals rely. They honestly believe that the processes of the school are readily transplanted to the hospital, and that what is common in the laboratory may be attempted in the ward. Yet we have a distinguished medical lecturer (Professor Oder) so indifferent to the conditions which govern maintenance by voluntary gift, and so unable to appraise them, as to state publicly that he "would like every student to undertake a small piece of research work." Not long ago, certain revelations concerning a too enthusiastic surgeon in Germany illustrated the possibilities, and people ought to be made aware that in English hospitals nothing stands between the patient and undue experimentalism but the personal character of the member of the staff in charge. This, happily, is all-sufficient in the vast majority of instances. In the case referred to, German practitioners were not slow to denounce the doings of their colleague, but the certainty of condemnation upon discovery did not render the delinquency impossible, nor safeguard the patients from its consequences.

